# Association between serum lipid levels over time and risk of Parkinson’s disease

**DOI:** 10.1038/s41598-022-25180-8

**Published:** 2022-12-05

**Authors:** Kyungduk Hurh, Minah Park, Sung-in Jang, Eun-Cheol Park, Suk-Yong Jang

**Affiliations:** 1 Department of Preventive Medicine, Yonsei University College of Medicine, Seoul, Republic of Korea; 2 Institute of Health Services Research, Yonsei University, Seoul, Republic of Korea; 3 Department of Public Health, Graduate School, Yonsei University, Seoul, Republic of Korea; 4 Department of Healthcare Management, Graduate School of Public Health, Yonsei University, 50-1 Yonsei-ro, Seodaemun-gu, Seoul, 03722 Republic of Korea

**Keywords:** Parkinson's disease, Dyslipidaemias, Risk factors

## Abstract

The role of serum lipids in Parkinson’s disease (PD) remains controversial. We aimed to evaluate the association between time-varying serum lipid levels and the risk of PD. This study included an assessment of the complete lipid profiles of 200,454 individuals from the 2002–2019 Korean National Health Insurance Health Screening Cohort. Time-dependent Cox proportional hazard regression models were used to evaluate the association between serum lipid levels over time and the risk of PD. Individuals in the lowest tertile of total cholesterol and low-density lipoprotein cholesterol had a 1.17 times [hazard ratio (HR) 1.17; 95% confidence interval (CI) 1.04–1.31] and 1.19 times (HR 1.19; 95% CI 1.06–1.34) higher risk of PD than those in middle tertile, respectively. Individuals in the highest high-density lipoprotein cholesterol tertile had a 0.89 times (HR 0.89; 95% CI 0.79–1.00) lower risk of PD than those in middle tertile, but the association was less robust in sensitivity analyses. Serum triglyceride levels were not related to the risk of PD. Our results suggest that the serum total and low-density lipoprotein cholesterol levels over time are inversely associated with the risk of PD. Further research is warranted to confirm these findings and reveal the underlying mechanisms.

## Introduction

Parkinson’s disease (PD) is the second most common neurodegenerative disease, with a lifetime risk of 2% among men and 1.3% among women^1^.

The pathological features of PD include the degeneration of dopaminergic neurons in the substantia nigra and widespread accumulation of α-synuclein^2,3^. Although the etiology of PD is not fully understood, epidemiologic studies have identified potential risk factors and protective factors for PD, including serum lipid levels^1^. However, studies investigating the association between serum lipid levels and PD risk have yielded inconsistent findings. A Finnish cohort study reported that individuals with higher serum total cholesterol (TC) levels had an increased risk of PD than those with lower TC level^4^. In a Korean cohort study, higher triglyceride (TG) and high-density lipoprotein cholesterol (HDL-C) levels were associated with an increased risk of PD^5^.

The risk of PD was not associated with history of diagnosed hypercholesterolemia in the Nurses’ Health Study and Health Professionals Follow-Up Study ^6^. In a Danish study using the Mendelian randomization method, no association was found between low-density lipoprotein cholesterol (LDL-C) and the risk of PD, whereas a Finnish study using a similar method reported that high levels of TC, TG, and LDL-C were related to a lower risk of PD^7,8^.

Other recent cohort and case–control studies reported that TC, LDL-C, HDL-C, and TG levels were inversely associated with the risk of PD^9–14^.

Heterogeneity among studies may be due to differences in methodology, including study design, sample size, and control for possible confounders. Although many studies had a prospective design, most did not reflect the time-varying nature of serum lipid levels over time. In addition, studies often overlooked the effect of potential confounders, such as statin use, lifestyle, and medical history, which were also thought to be related to the risk of PD. Finally, there is limited longitudinal evidence on the relationship between blood lipid levels and the risk of PD in Asian populations.

Therefore, this study aimed to investigate the association between time-varying serum lipid levels (TC, LDL-C, HDL-C, and TG) and the incidence of PD using Korean population-based data.

## Results

**Figure 1:**
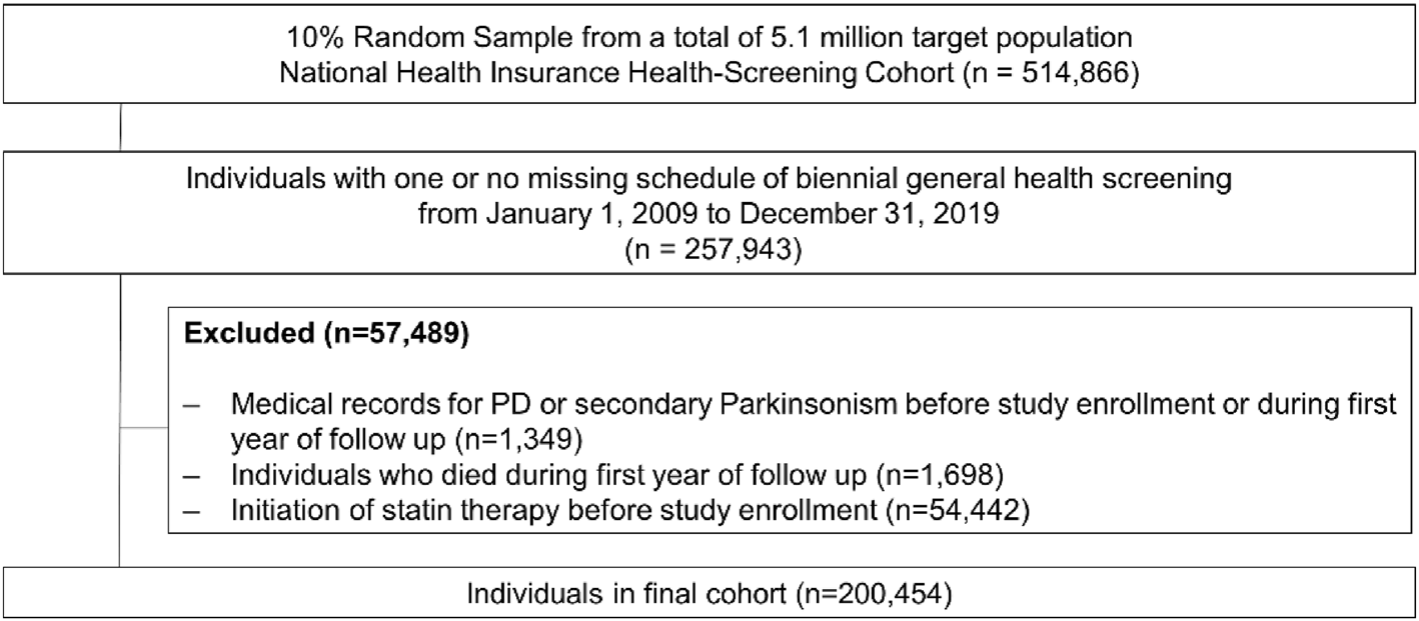
Flowchart of participant selection. PD, Parkinson’s disease.

Between January 1, 2002, and December 31, 2019, 257,943 individuals met the inclusion criteria for our study. Individuals with a medical record of PD or secondary Parkinsonism before or during the first year of follow-up (n = 1,349), those who died during the first year of follow-up (n = 1698), and those with statin prescriptions (n = 54,442) prior to the first health screening were excluded. The final sample included 200,454 individuals (Fig. 1).

The mean and cumulative follow-up time were 8.5 years and 1,713,530 person-years, respectively, and 1712 PD cases were identified during the study period. The mean age of the participants at baseline was 57.4 (± 8.6) years and 113,577 (56.7%) of the participants were male. The mean serum lipid levels at baseline were 198.7 (± 34.4) mg/dL for TC, 118.6 (± 35.6) mg/dL for LDL-C, 54.8 (± 23.9) mg/dL for HDL-C, and 131.4 (± 81.6) mg/dL for TG. A total of 41,851 (20.9%) individuals started statins after enrollment in the study (Table 1).

**Table 1:** General characteristics of the study population.

Characteristic	Total (n = 200,454)	
Total cholesterol levels at baseline, mg/dL (Mean, SD)	198.7	(34.4)
LDL cholesterol levels at baseline, mg/dL (Mean, SD)	118.6	(35.6)
HDL cholesterol levels at baseline, mg/dL (Mean, SD)	54.8	(23.9)
Triglyceride levels at baseline, mg/dL (Mean, SD)	131.4	(81.6)
Age at baseline, years (Mean, SD)	57.4	(8.6)
**Sex (No, %)**		
Male	113,577	(56.7)
Female	86,877	(43.3)
**Residential area (No, %)**		
Urban	89,661	(44.7)
Rural	110,793	(55.3)
**Smoking status (No, %)**		
Never-smoker	125,002	(62.4)
Ex-smoker	37,161	(18.5)
Current-smoker	34,431	(17.2)
Unspecified	3858	(1.9)
**Alcohol consumption, units/weeks (No, %)**		
None	22,126	(11.0)
≤ 7	120,884	(60.3)
8–14	20,914	(10.4)
> 15	36,530	(18.2)
**Physical activity, METs∙min/weeks (No, %)**		
0–499	58,774	(29.3)
500–999	61,008	(30.4)
≥ 1000	80,672	(40.2)
**BMI, kg/m**^**2**^** (No, %)**		
< 25	138,052	(68.9)
≥ 25	62,336	(31.1)
Unspecified	66	(0.0)
**History of stroke (No, %)**		
No	197,097	(98.3)
Yes	3357	(1.7)
**History of diabetes mellitus (No, %)**		
No	179,855	(89.7)
Yes	20,599	(10.3)
**History of hypertension (No, %)**		
No	131,515	(65.6)
Yes	68,939	(34.4)
**Statin usage after study enrollment**		
No	158,603	(79.1)
Yes	41,851	(20.9)
Parkinson’s disease cases	1712	(0.9)
Person years	1,713,530	
Mean follow-up time, years	8.5	

SD, standard deviation; No, number; LDL, low-density lipoprotein; HDL, high-density lipoprotein.

The crude incidence rates of PD (per 100,000 person-years) among individuals in the lowest, middle, and highest tertiles of each serum lipid fraction were 128.9, 88.3, and 80.4 for TC, 125.3. 89.7, and 83.9 for LDL-C, 115.5, 103.8, and 83.9 for HDL-C, 100.7, 105.6, and 93.1 for TG, respectively. After adjusting for all covariates, individuals in the lowest TC tertile had a 1.17 times [hazard ration (HR) 1.17; 95% confidence interval (CI) 1.04–1.31] higher risk of PD than those in the middle TC tertile. Individuals in the highest TC tertile showed no statistically significant difference (HR 1.03; 95% CI 0.91–1.17) in the risk of PD, compared with those in the middle TC tertiles. Compared to individuals with middle LDL-C tertile, those with lowest LDL-C tertile had 1.19 times (HR 1.19; 95% CI 1.06–1.34) higher risk of PD, after adjusting all covariates. Individuals in the highest and middle LDL-C tertiles did not show a difference in the risk of PD (HR 1.06; 95% CI 0.94–1.20). Compared to individuals in the middle HDL-C tertile, those in the lowest HDL-C tertile did not show a difference in the risk of PD (HR 1.01; 95% CI 0.90–1.14), while those in the highest HDL-C tertile had a 0.89 times (HR 0.89; 95% CI 0.79–1.00) lower risk of PD. Serum TG level was not associated with the risk of developing PD (Table 2).

**Table 2:** Association between serum lipid levels over time and risk of Parkinson’s disease.

Variables	Events	PY	Crude IR (per 100,000 PY)	Adjusted HR (95% CI)^a^		
				Model 1^b^	Model 2^c^	Model 3^d^
**Total cholesterol levels (tertiles)**						
Lowest	775	601,113	128.9	1.19 (1.06–1.33)**	1.16 (1.04–1.30)*	1.17 (1.04–1.31)**
Middle	474	536,784	88.3	**1.00**	**1.00**	**1.00**
Highest	463	575,633	80.4	1.02 (0.90–1.16)	1.03 (0.90–1.17)	1.03 (0.91–1.17)
**LDL cholesterol levels (tertiles)**						
Lowest	734	585,733	125.3	1.21 (1.08–1.36)***	1.19 (1.06–1.33)**	1.19 (1.06–1.34)**
Middle	489	545,129	89.7	**1.00**	**1.00**	**1.00**
Highest	489	582,668	83.9	1.05 (0.93–1.19)	1.06 (0.94–1.20)	1.06 (0.94–1.20)
**HDL cholesterol levels (tertiles)**						
Lowest	556	481,559	115.5	1.03 (0.92–1.15)	1.01 (0.90–1.14)	1.01 (0.90–1.14)
Middle	640	616,815	103.8	**1.00**	**1.00**	**1.00**
Highest	516	615,156	83.9	0.88 (0.78–0.99)*	0.89 (0.79–1.00)*	0.89 (0.79–1.00)*
**Triglyceride levels (tertiles)**						
Lowest	591	586,959	100.7	1.00 (0.89–1.12)	1.00 (0.89–1.12)	1.00 (0.90–1.13)
Middle	606	573,670	105.6	**1.00**	**1.00**	**1.00**
Highest	515	552,901	93.1	0.99 (0.88–1.11)	0.98 (0.88–1.11)	0.99 (0.88–1.11)

Abbreviations: IR, incidence rate; PY, person years; HR, hazard ratio; CI, confidence interval; LDL, low-density lipoprotein; HDL, high-density lipoprotein.

^a^For each blood lipid fraction, separate time-dependent Cox regression models was fitted.

^b^Adjusted for age, sex, area of residence, smoking status, alcohol consumption, levels of physical activity, and BMI.

^c^Adjusted for age, sex, area of residence, smoking status, alcohol consumption, levels of physical activity, BMI, and past medical history (stroke, diabetes mellitus, and hypertension).

^d^Adjusted for age, sex, area of residence, smoking status, alcohol consumption, levels of physical activity, BMI, past medical history (stroke, diabetes mellitus, and hypertension), and cumulative duration of statin usage.

***p < 0.001; **p < 0.01; *p < 0.05.

Sensitivity analyses showed results similar to those of the main analysis of serum TC and LDL-C levels. However, only two of the four sensitivity analyses showed statistical significance for serum HDL-C levels, although the directions of association were the same as those in the main analysis (Tables 3 and 4). Among 1712 patients with PD, the risk of dementia in PD was related to serum TC, HDL-C, and TG levels (although the directions of the association were not uniform) whereas PD mortality was not associated with any serum lipid level (Supplementary Table S1 and S2).

**Table 3:** Results of sensitivity analyses using alternative diagnostic criteria for PD.

Variables	Events	PY	Crude IR (per 100,000 PY)	Adjusted HR (95% CI)^a^		
				Model 1^b^	Model 2^c^	Model 3^d^
**Antiparkinsonian drug and ≥ 1 ICD-10 code**						
**Total cholesterol levels (tertiles)**						
Lowest	884	599,561	147.4	1.15 (1.04–1.28)*	1.13 (1.02–1.26)*	1.13 (1.02–1.25)*
Middle	589	535,763	109.9	**1.00**	**1.00**	**1.00**
Highest	584	574,552	101.6	1.02 (0.91–1.14)	1.03 (0.92–1.15)	1.03 (0.92–1.16)
**LDL cholesterol levels (tertiles)**						
Lowest	845	584,294	144.6	1.14 (1.03–1.27)*	1.13 (1.01–1.25)*	1.12 (1.01–1.24)*
Middle	623	544,029	114.5	**1.00**	**1.00**	**1.00**
Highest	589	581,553	101.3	0.97 (0.86–1.08)	0.98 (0.98–1.09)	0.98 (0.87–1.10)
**HDL cholesterol levels (tertiles)**						
Lowest	661	480,304	137.6	1.01 (0.91–1.13)	1.00 (0.90–1.11)	1.00 (0.90–1.11)
Middle	763	615,496	124.0	**1.00**	**1.00**	**1.00**
Highest	633	614,076	103.1	0.89 (0.81–0.99)*	0.89 (0.81–1.00)*	0.91 (0.82–1.01)
**Triglyceride levels (tertiles)**						
Lowest	680	585,809	116.1	0.98 (0.88–1.08)	1.00 (0.88–1.09)	1.00 (0.89–1.10)
Middle	736	572,340	128.6	**1.00**	**1.00**	**1.00**
Highest	641	551,726	116.2	0.99 (0.89–1.10)	0.98 (0.88–1.09)	0.99 (0.89–1.10)
**≥ 1 admission or ≥ 2 ambulatory visits with PD, and ≥ 1 PD diagnosis by a neurologist**						
**Total cholesterol levels (tertiles)**						
Lowest	645	606,030	106.4	1.19 (1.05–1.35)**	1.17 (1.03–1.33)*	1.17 (1.03–1.33)*
Middle	395	539,618	73.2	**1.00**	**1.00**	**1.00**
Highest	379	579,933	65.4	1.00 (0.87–1.15)	1.01 (0.87–1.16)	1.01 (0.88–1.16)
**LDL cholesterol levels (tertiles)**						
Lowest	625	590,833	105.8	1.30 (1.14–1.47)***	1.28 (1.13–1.45)***	1.27 (1.12–1.44)***
Middle	392	548,036	71.5	**1.00**	**1.00**	**1.00**
Highest	402	586,711	68.5	1.07 (0.93–1.23)	1.07 (0.93–1.23)	1.08 (0.94–1.24)
**HDL cholesterol levels (tertiles)**						
Lowest	467	485,247	96.2	1.02 (0.90–1.16)	1.01 (0.89–1.15)	1.01 (0.89–1.15)
Middle	528	621,153	85.0	**1.00**	**1.00**	**1.00**
Highest	424	619,179	68.5	0.87 (0.76–0.99)*	0.87 (0.87–0.99)*	0.88 (0.77–1.00)*
**Triglyceride levels (tertiles)**						
Lowest	482	590,083	81.7	0.98 (0.88–1.08)	0.99 (0.87–1.12)	0.99 (0.87–1.12)
Middle	499	577,624	86.4	**1.00**	**1.00**	**1.00**
Highest	438	557,873	78.5	0.99 (0.89–1.10)	1.01 (0.89–1.15)	1.02 (0.89–1.16)
** ≥ 1 admission or ≥ 2 ambulatory visits with extended definition of PD**^**e**^						
**Total cholesterol levels (tertiles)**						
Lowest	1057	603,531	175.1	1.18 (1.07–1.30)***	1.16 (1.05–1.28)**	1.15 (1.04–1.27)**
Middle	663	538,077	123,2	**1.00**	**1.00**	**1.00**
Highest	642	578,481	111.0	1.00 (0.90–1.11)	1.01 (0.90–1.12)	1.01 (0.91–1.13)
**LDL cholesterol levels (tertiles)**						
Lowest	1012	588,433	172.0	1.24 (1.22–1.37)***	1.22 (1.10–1.34)***	1.20 (1.09–1.33)***
Middle	670	546,433	122.6	**1.00**	**1.00**	**1.00**
Highest	680	585,223	116.2	1.04 (0.94–1.16)	1.05 (0.95–1.17)	1.06 (0.95–1.18)
**HDL cholesterol levels (tertiles)**						
Lowest	769	483,438	159.1	1.02 (0.93–1.12)	1.01 (0.91–1.11)	1.01 (0.91–1.11)
Middle	873	619,174	141.0	**1.00**	**1.00**	**1.00**
Highest	720	617,477	116.6	0.89 (0.81–0.99)*	0.90 (0.82–0.99)*	0.90 (0.82–1.00)*
**Triglyceride levels (tertiles)**						
Lowest	797	588,259	135.5	0.95 (0.87–1.05)	0.96 (0.87–1.06)	0.96 (0.87–1.06)
Middle	860	575,682	149.4	**1.00**	**1.00**	**1.00**
Highest	705	55,6147	126.8	0.94 (0.85–1.04)	0.94 (0.85–1.03)	0.94 (0.85–1.04)

IR, incidence rate; PY, person years; HR, hazard ratio; CI, confidence interval; LDL, low-density lipoprotein; HDL, high-density lipoprotein; PD, Parkinson’s disease.

^a^For each blood lipid fraction, separate time-dependent Cox regression models was fitted.

^b^Adjusted for age, sex, area of residence, smoking status, alcohol consumption, levels of physical activity, and BMI.

^c^Adjusted for age, sex, area of residence, smoking status, alcohol consumption, levels of physical activity, BMI, and past medical history (stroke, diabetes mellitus, and hypertension).

^d^Adjusted for age, sex, area of residence, smoking status, alcohol consumption, levels of physical activity, BMI, past medical history (stroke, diabetes mellitus, and hypertension), and cumulative duration of statin usage.

^e^Parkinson’s disease (ICD G20), other forms of secondary parkinsonism (ICD-10 codes G21 and G22), other degenerative diseases in basal ganglia (ICD-10 code G23), and movement disorders in diseases classified elsewhere (ICD-10 code G26).

***p < 0.001; **p < 0.01; *p < 0.05.

**Table 4:** Results of sensitivity analyses using 3-year time lag.

Variables	Events	PY	Crude IR (per 100,000 PY)	Adjusted HR (95% CI)^a^		
				Model 1^b^	Model 2^c^	Model 3^d^
**Total cholesterol levels (tertiles)**						
Lowest	549	407,160	134.8	1.32 (1.15–1.52)***	1.31 (1.14–1.50)***	1.31 (1.14–1.50)***
Middle	342	392,455	87.1	**1.00**	**1.00**	**1.00**
Highest	337	377,782	89.2	1.14 (0.98–1.32)	1.14 (0.98–1.33)	1.14 (0.98–1.33)
**LDL cholesterol levels (tertiles)**						
Lowest	571	436,487	130.8	1.39 (1.22–1.57)***	1.30 (1.14–1.47)**	1.21 (1.06–1.37)**
Middle	407	462,694	95.4	**1.00**	**1.00**	**1.00**
Highest	442	452,992	97.6	1.00 (0.88–1.15)	1.04 (0.91–1.19)	1.14 (0.99–1.33)
**HDL cholesterol levels (tertiles)**						
Lowest	477	371,419	128.4	1.20 (1.06–1.36)**	1.14 (1.01–1.29)*	1.07 (0.94–1.21)
Middle	519	476,985	108.8	**1.00**	**1.00**	**1.00**
Highest	424	467,769	90.6	0.81 (0.71–0.92)**	0.85 (0.74–0.96)*	0.90 (0.79–1.03)
**Triglyceride levels (tertiles)**						
Lowest	501	488,265	111.8	1.08 (0.96–1.23)	1.09 (0.96–1.24)	1.09 (0.96–1.24)
Middle	480	438,474	109.4	**1.00**	**1.00**	**1.00**
Highest	439	429,434	102.2	1.03 (0.90–1.17)	1.02 (0.89–1.15)	1.02 (0.90–1.16)

IR, incidence rate; PY, person years; HR, hazard ratio; CI, confidence interval; LDL, low-density lipoprotein; HDL, high-density lipoprotein; PD, Parkinson’s disease.

^a^For each blood lipid fraction, separate time-dependent Cox regression models was fitted.

^b^Adjusted for age, sex, area of residence, smoking status, alcohol consumption, levels of physical activity, and BMI.

^c^Adjusted for age, sex, area of residence, smoking status, alcohol consumption, levels of physical activity, BMI, and past medical history (stroke, diabetes mellitus, and hypertension).

^d^Adjusted for age, sex, area of residence, smoking status, alcohol consumption, levels of physical activity, BMI, past medical history (stroke, diabetes mellitus, and hypertension), and cumulative duration of statin usage.

***p < 0.001; **p < 0.01; *p < 0.05.

## Discussion

IN this population-based cohort study, we found that lower serum TC and LDL-C levels were associated with an increased risk of PD, whereas higher serum HDL-C levels were associated with a decreased risk of PD, even after adjusting for potential confounders, including statin use. However, serum TG levels were not related to PD risk in our study. In the sensitivity analyses, the association between serum HDL-C level and PD was less robust, while other findings were similar to those of the main analysis.

Our results are consistent with findings from previous studies—serum TC, LDL-C, and HDL-C were inversely associated with the risk of PD^8,9,13^; individuals with low cholesterol intake had an increased risk of PD^15,16^, and high TC levels were related to the slow progression of PD symptoms^17^. Our findings provide longitudinal evidence for the association between serum lipid levels and PD risk in the Asian population. Moreover, we assessed repeated measurements of serum lipid levels over time and performed sensitivity analyses to assess the robustness of the relationship between serum lipid levels and PD risk.

In the present study, the impact of serum cholesterol levels on the risk of PD was small or modest compared to previous studies that reported that the risk of PD differed by up to two-fold according to the serum cholesterol level^11,13^. Potential confounders, such as lifestyle factors, comorbidities, and statin use, which were often neglected in other studies, might have contributed to this gap. Furthermore, we found that an altered serum lipid profile may be related to the development of dementia after PD diagnosis, although the findings were preliminary. The lack of association between PD-mortality and serum lipid levels might imply that an altered lipid profile was possibly related to a cognitive function independently of other conditions, such as nutritional status.

The underlying mechanisms of the findings of the present study are unclear and require further research. Elevated cholesterol levels might be related to protective factors of PD (e.g., increased serum cholesterol due to smoking and physical activity after dyslipidemia diagnosis)^1^. In addition, neurodegenerative changes prior to the diagnosis of PD may have led to lifestyle changes that are linked to cholesterol changes.

As suggested in literature, dysregulation of cholesterol metabolism may be related to neurodegenerative processes in the brain^8,9^. Cholesterol is an essential component of cell membranes and myelin sheaths, and maintaining balanced cholesterol regulation is important for neuronal signaling and synaptic function^18–20^. The brain contains the highest levels of cholesterol in the body, and alterations in cholesterol homeostasis and biosynthesis can cause neurodegeneration in the central nervous system (CNS)^21^. In an animal model, mice with cholesterol synthesis deficiency developed motor symptoms, such as tremor and ataxia^22^. Epidemiological studies have found that abnormal serum cholesterol levels are also associated with other neurodegenerative disorders such as Alzheimer’s disease (AD), Huntington’s disease (HD), and amyotrophic lateral sclerosis^23–26^.

However, since it is difficult for lipoprotein-bound cholesterol to cross the blood–brain barrier (BBB), it is difficult to say that alterations in serum cholesterol levels directly affect cholesterol levels in CNS^27,28^.

Recent studies have found a shared genetic etiology between serum cholesterol levels and PD, as reported in other neurodegenerative disease such as AD and HD^25,26,29^. Analyses of genome-wide association studies data revealed that dysregulation in lipid metabolism is associated with molecular processes that are considered important in PD development, such as oxidative stress, endoplasmic reticulum stress response, and immune response^29–31^. Combined with the difficulty of lipoprotein crossing the BBB, these findings suggest that serum cholesterol levels and incidence of PD may be indirectly related through genetic pathways.

Our study had several limitations. First, PD diagnosis could be imprecise because we identified PD cases using the ICD-10 codes. Therefore, we tried to improve diagnostic accuracy by defining PD cases as those with at least one admission or two outpatient visits for PD, and performed sensitivity analysis with alternative PD definitions. However, there was still a possibility of missing or misdiagnosed cases due to lack of clinical case certification and individual chart review. The number of patients with PD might have been underestimated, because our PD definition (including sensitivity analyses) did not include the patients with dystonia or other movement disorders (ICD-10 codes G24 and G25), who might have actually had idiopathic PD. Second, we were unable to classify patients with PD genetically (mutations in *LRRK2, GBA, or Parkin*) due to lack of relevant data. Third, although we adjusted for several potential confounders, residual confounding could be present because information on environmental exposure, genetic factors, dietary factors, and caffeine intake were not available in our data. Fourth, because PD has a long clinical course, and the onset of PD symptoms may precede the diagnosis of PD, the possibility of reverse causation (e.g., low cholesterol levels as a result of PD progression) cannot be excluded. However, our study had a relatively long washout period (minimum 7 years), and our setting with 1-year and 3-year time lags could reduce the possibility of reverse causation. Therefore, future studies including detailed clinical and genetic information on PD are required to verify the findings of the present study.

In conclusion, based on time-varying serum lipids levels, our study suggests that serum TC, LDL-C levels may be inversely associated with the risk of PD after controlling potential confounders. However, further studies are warranted to elucidate underlying mechanisms and specify the optimal range of serum cholesterol levels to minimize the risk of PD.

## Methods

### Study subjects and data sources

Data for this study were obtained from the 2002–2019 Korea National Health Insurance Service Health Screening Cohort (NHIS-HEALS). The National Health Insurance Service (NHIS), a single-payer universal health insurance program, provides a general health-screening program for all Korean citizens aged ≥ 40 years every 2 years^32^. In 2009, the Korean general health screening program started measuring the full lipid profiles (TC, LDL-C, HDL-C, and TG) of participants. The NHIS-HEALS comprised of 514,866 participants in a general health-screening program, aged between 40 and 79 years in 2002, which is a 10% simple random sample of the target population^32^. The NHIS-HEALS includes anonymized participant information (demographics, medical records, and health screening database), and the participants were followed up until their loss of eligibility due to death or emigration^33^.

To fully address time-varying serum lipid levels, only individuals with ≤ 1 missing schedule of biennial health screening were included in the study. Individuals who used statins before study enrollment were excluded to eliminate the potential confounding effects of statins.

This study was approved by the Institutional Review Board of Yonsei University Health System (IRB No: 4-2022-0863) and adhered to the tenets of the Declaration of Helsinki. Written informed consent was waived by Institutional Review Board of Yonsei University Health System (IRB No: 4-2022-0863) because of the retrospective nature of the study and NHIS-HEALS contains anonymized data.

### PD identification

Incident PD cases, the outcome of study, were identified based on the International Statistical Classification of Diseases (ICD-10) code of PD (G20). To reduce false-positive cases and ensure diagnostic validity, individuals with at least two outpatient visits or one admission for PD were classified as patients with PD. Individuals with a medical record of PD or secondary Parkinsonism before the index date were excluded from the study, allowing a minimum washout period of 7 years (2002–2008).

### Measurement of lipid profiles and covariates

The Korean general health-screening program has been measuring the serum lipid levels (TC, LDL-C, HDL-C, and TG) of participants every two years since 2009. Serum lipid levels were categorized into tertiles and were included as time-dependent variables. The cutoff values for categorizing each lipid profile were based on values of the first measurements (mg/dL, TC, lowest ≤ 182, middle 183–210, highest ≥ 211; LDL-C, lowest ≤ 103, middle 104–129, highest ≥ 130; HDL-C lowest ≤ 45, middle 46–57, highest ≥ 58; TG low ≤ 88, middle 88–138, highest ≥ 139).

Although we excluded individuals who had used statins before the study period, some participants initiated statin therapy during the study period. Thus, the cumulative duration of statin use (none, < 1 year, 1–2 years, > 2 years) corresponding to the lipid-level-based time segment was included as a covariate. Other potential confounders were identified during study enrollment. Age (continuous variable),sex, area of residence (urban or rural), smoking status (never smoker, ex-smoker, current smoker, or unspecified), alcohol consumption (none, ≤ 7, 7–14, or > 14 units per week), levels of physical activity (0–499, 500–999, or > 1000 MET∙ min per week), body mass index (< 25, ≥ 25 kg/m^2^, or unspecified), and past medical history (stroke, diabetes mellitus, and hypertension, individuals with more than two outpatient visits or one admission over three years before the index date, based on ICD-10 codes) were included as covariates.

### Sensitivity analyses

Four additional sensitivity analyses were performed. First, the definition of PD was changed to include participants who used antiparkinsonian drugs (levodopa, levodopa combined with decarboxylase inhibitor or catechol-O-methyl transferase inhibitor, dopamine agonists, monoamine oxidase B inhibitors, amantadine, and anticholinergics) with one or more ICD-10 codes. Second, we changed the definition of PD to include individuals with ≥ 1 hospital admission or ≥ 2 ambulatory visits for PD, and ≥ 1 PD diagnosis made by a neurologist. Third, to assess potential underestimation of PD cases, we used extended definition of PD to include individuals with other forms of secondary parkinsonism (ICD-10 codes G21 and G22), other degenerative diseases in basal ganglia (ICD-10 code G23), and movement disorders in diseases classified elsewhere (ICD-10 code G26). Fourth, the 1-year time lag was replaced by a 3-year time lag to reflect the PD diagnosis.

### Statistical analyses

The baseline characteristics of participants were described as mean and standard deviation for continuous variables, and the number and percentage of participants for categorical variables. To reflect the time from PD onset to diagnosis and reduce the possibility of reverse causation, all analyses were performed with a 1-year time lag.

The association between serum lipid levels and the risk of PD was evaluated using a time-dependent Cox proportional hazard regression, and the effect size was estimated as the HR. In addition, the crude incidence rate (number of PD cases per 100,000 person-years) for each serum lipid level was calculated. The middle tertile for each serum lipid level was used as the reference value. The index date was the date of the first blood test, and any changes in the lipid tertiles over time were updated as separate time segments. The participants were followed up until PD development, death, or December 31, 2019. Among individuals who developed PD during follow-up, we performed preliminary study to evaluate whether serum lipid levels over time are related to PD complications, such as dementia in PD (ICD-10 code F023) or death due to PD. Statistical significance was defined as a two-sided *p* value < 0.05. All analyses were performed using the SAS Enterprise Guide software (version 7.1; SAS Institute, Cary, NC, USA).

## Supplementary Information

Supplementary Information.

## Data Availability

The datasets generated and/or analyzed during the current study are not publicly available due because the NHIS exclusively allows authorized persons to access data in a separate space. Upon an individual researcher's data set request, NHIS provides customized data to the researcher from the National Health Insurance Sharing Service at https://nhiss.nhis.or.kr/bd/ab/bdabb006iv.do.
