# Supplementary material for: Association between serum lipid levels over time and risk of Parkinson’s disease
Source: Sci Rep. 2022 Dec 5;12:21020. doi: 10.1038/s41598-022-25180-8 (PMC9722928; doi:10.1038/s41598-022-25180-8)
Supplement: Supplementary file 1 — Supplementary Information. [file 41598_2022_25180_MOESM1_ESM.docx]

**Supplementary Materials**

**Table S1.** Association between serum lipid levels and risk of dementia in PD among patients with PD

**Table S2.** Association between serum lipid levels and PD mortality among patients with PD

**Table S3.** Pseudo R-squared values of each model in main analyses

Abbreviations: PD, Parkinson’s disease

| **Variables** | | **Events** | **Person years** | **Crude incidence rate (per 1000 person years)** | **Adjusted hazard ratio^b^ (95% CI)** |
| --- | --- | --- | --- | --- | --- |
| **Total cholesterol levels (tertiles)** | |  |  |  |  |
|  | lowest | 69 | 4,318 | 16.0 | 1.31 (0.83–2.06) |
|  | middle | 30 | 2,358 | 12.7 | **1.00** |
|  | highest | 35 | 1,930 | 18.1 | 1.71 (1.03–2.84) |
| **LDL cholesterol levels (tertiles)** | |  |  |  |  |
|  | lowest | 58 | 4,015 | 14.4 | 0.81 (0.53–1.22) |
|  | middle | 42 | 2,522 | 16.7 | **1.00** |
|  | highest | 34 | 2,068 | 16.4 | 1.00 (0.63–1.60) |
| **HDL cholesterol levels (tertiles)** | |  |  |  |  |
|  | lowest | 34 | 3,011 | 11.3 | 0.56 (0.36–0.87) |
|  | middle | 55 | 2,970 | 18.5 | **1.00** |
|  | highest | 45 | 2,624 | 17.1 | 1.12 (0.74–1.67) |
| **Triglyceride levels (tertiles)** | |  |  |  |  |
|  | lowest | 56 | 3,132 | 17.9 | 1.15 (0.78–1.71) |
|  | middle | 51 | 3,043 | 16.8 | **1.00** |
|  | highest | 27 | 2,431 | 11.1 | 0.59 (0.37–0.95) |
| ^a^For each serum lipid fraction, separate time-dependent Cox regression models was fitted | | | | | |
| ^b^Adjusted for age, sex, area of residence, smoking status, alcohol consumption, levels of physical activity, BMI, past medical history (stroke, diabetes mellitus, and hypertension), and cumulative duration of statin usage | | | | | |
| PD, Parkinson's disease | | | | | |

**Table S1. Association between serum lipid levels and risk of dementia in PD among patients with PD^a^**

The analysis was performed among 1,712 individuals who developed Parkinson’s diseases (PD). The last measurement of serum lipid levels before diagnosis of PD and their changes over time was included as exposure (same as main analysis). The covariates and statistical methods were the same as in the main analysis. The analysis should be considered as a preliminary one, because low participation in health screening after PD development, small sample size, and lack of information on potential confounders (including educational levels).

**Table S2. Association between serum lipid levels and PD mortality among patients with PD^a^**

| **Variables** | | **Events** | **Person years** | **Crude incidence rate (per 1000 person years)** | **Adjusted hazard ratio^b^ (95% CI)** |
| --- | --- | --- | --- | --- | --- |
| **Total cholesterol levels (tertiles)** | |  |  |  |  |
|  | lowest | 122 | 4,460 | 27.4 | 1.39 (0.97–1.99) |
|  | middle | 41 | 2,452 | 16.7 | **1.00** |
|  | highest | 43 | 2,007 | 21.4 | 1.42 (0.92–2.19) |
| **LDL cholesterol levels (tertiles)** | |  |  |  |  |
|  | lowest | 107 | 4,127 | 25.9 | 0.96 (0.70–1.32) |
|  | middle | 60 | 2,630 | 22.8 | **1.00** |
|  | highest | 42 | 2,160 | 19.4 | 0.90 (0.60–1.60) |
| **HDL cholesterol levels (tertiles)** | |  |  |  |  |
|  | lowest | 73 | 3,105 | 23.5 | 0.88 (0.63–1.22) |
|  | middle | 77 | 3,083 | 25.0 | **1.00** |
|  | highest | 63 | 2,730 | 23.1 | 0.99 (0.71–1.39) |
| **Triglyceride levels (tertiles)** | |  |  |  |  |
|  | lowest | 92 | 3,223 | 28.5 | 1.30 (0.95–1.79) |
|  | middle | 71 | 3,199 | 22.2 | **1.00** |
|  | highest | 53 | 2,496 | 21.2 | 0.98 (0.68–1.41) |
| ^a^For each blood lipid fraction, separate time-dependent Cox regression models was fitted | | | | | |
| ^b^Adjusted for age, sex, income, registered disability, area of residence, smoking status, alcohol consumption, levels of physical activity, BMI, Charlson comorbidity index, and cumulative duration of statin usage | | | | | |
| PD, Parkinson's disease | | | | | |

The past medical histories (stroke, diabetes, and hypertension) among covariates were replaced with the Charlson comorbidity index to explain mortality of individuals.

**Table S3. Pseudo R-squared values of each model in main analyses**

| **Models** | **Pseudo R^2^** | | |
| --- | --- | --- | --- |
|  | **Model 1** | **Model 2** | **Model 3** |
| **TC** | 4.23% | 4.29% | 4.31% |
| **LDL-C** | 4.25% | 4.28% | 4.31% |
| **HDL-C** | 4.22% | 4.28% | 4.31% |
| **TG** | 4.20% | 4.26% | 4.27% |

Model 1 includes age, sex, area of residence, smoking status, alcohol consumption, levels of physical activity, and BMI as covariates.

Model 2 includes age, sex, area of residence, smoking status, alcohol consumption, levels of physical activity, BMI, and past medical history (stroke, diabetes mellitus, and hypertension) as covariates.

Model 3 includes age, sex, area of residence, smoking status, alcohol consumption, levels of physical activity, BMI, past medical history (stroke, diabetes mellitus, and hypertension), and cumulative duration of statin usage as covariates.

The low pseudo R-squared values are the results of large portion of censored data in our model (because of low incidence of Parkinson’s disease and time-varying segment for the serum lipid levels)^1^.

**References**

1. Hosmer, D. W., and Lemeshow S. Applied Survival Analysis: Regression Modeling of Time to Event Data. 228–230 (John Wiley and Sons Inc, 1999).
